# The Effect on Flexibility and a Variety of Performance Tests of the Addition of 4 Weeks of Soleus Stretching to a Regular Dynamic Stretching Routine in Amateur Female Soccer Players

**DOI:** 10.3390/sports11070138

**Published:** 2023-07-19

**Authors:** Mohammad Alimoradi, Mansour Sahebozamani, Elham Hosseini, Andreas Konrad, Sajad Noorian

**Affiliations:** 1 Department of Sports Injuries and Corrective Exercises, Faculty of Sports Sciences, Shahid Bahonar University, Kerman 76169-14111, Iran; malimoradi@sport.uk.ac.ir (M.A.); sahebozamani@uk.ac.ir (M.S.); hosseinielham7400@sport.uk.ac.ir (E.H.); 2 Institute of Human Movement Science, Sport and Health, University of Graz, 8010 Graz, Austria; 3 Department of Statistics, Faculty of Science, University of Qom, Qom 37161-46611, Iran; sajad.noorian@gmail.com

**Keywords:** soccer, muscle stretching, soleus muscle, range of motion, balance, performance

## Abstract

The purpose of this study was to investigate the effects of 4 weeks of soleus stretching on ankle flexibility and dynamic balance, as well as selected monitoring and performance tests in soccer. Forty-five healthy female soccer players were randomly divided into a regular stretching group, a regular stretching group with soleus stretching, and a control group. Dynamic stretching protocols were performed for 4 weeks during three sessions per week as part of routine exercises. The regular group stretched three muscle groups (i.e., gastrocnemius, quadriceps, and hamstrings), while the regular + soleus group also stretched the soleus muscle. Before and after the stretching intervention, the ankle range of motion test, Y-balance test, drop jump test, dynamic knee valgus test, and Illinois Agility Running Test were performed. Ankle ROM, Y-balance, and DJ significantly improved in both intervention groups compared to controls. Only the regular + soleus group showed improvement in the Illinois Agility Running Test. Additionally, athletes performing the additional soleus stretching had greater improvements in ankle ROM and DJ but not in DKV or Y-balance. The results showed that adding soleus stretching into regular protocols can provide benefits for female soccer players in terms of performance parameters.

## 1. Introduction

Soccer is a sport that offers numerous physical benefits to female athletes [1]. However, previous studies have reported a high incidence of injury among this population, with nearly 4.6 injuries per 1000 training hours and 22.4 injuries per 1000 match hours. This high prevalence of injury is likely due to the repetitive movements at the end range of motion (ROM) in the lower limbs [2,3,4,5]. Therefore, athletes must maintain high fitness levels for these demands to minimize the risk of injury [6,7]. Various warm-up strategies, such as static stretching, dynamic stretching, or an integration of dynamic and static stretching, have been recommended [6]. Dynamic warm-ups (DWUs) have many positive effects on soccer-related attributes, such as ROM [4], vertical jump [8], sprinting [8], ball controlling, and kicking accuracy [9,10]. Among various warm-up protocols, most studies recommend DWUs as preferable to prepare athletes during the pre-activity phase [11,12]. Earlier evidence has shown that dynamic stretching (DS) prepares athletes to perform the demands of subsequent activity by improving ROM, the length of musculotendinous units, and mechanoreceptor reflexes [13]. Nevertheless, no consensus was found among studies regarding motor-task performances [14,15]. Regarding assessment tests performed to evaluate the quality of movements and identify athletes who are susceptible to injury, previous studies have shown that utilizing DWUs to prepare athletes before training and competition is beneficial for improving scores in tests and reducing the risk of injury [6,16,17]. However, in soccer, few studies have investigated the effect of DWUs on monitoring tests (MTs) [18,19]. Monitoring tests are standardized and repeatable profile tests, such as the hop test, dynamic knee valgus test, other battery tests, and force-plate jumps [20]. While there is evidence that a single dynamic stretching protocol can produce an immediate positive effect on muscle function, much less is currently understood about the long-term impact of repeating such warm-up protocols.

It has been demonstrated that dynamic stretching (DS) for lower limb muscles, including the gluteus, hamstrings, quadriceps, and calf muscles, is more effective than no stretching or static stretching (SS) in enhancing joint flexibility and athletic performance [4,8,13]. Multiple protocols have been performed on lower limbs. However, few studies have focused on investigating the effect of calf-muscle stretching in soccer players, and many have neglected to consider the impact of soleus-muscle stretching [9,21,22,23,24]. Since ankle flexibility is closely related to dynamic balance [25], which is a key component of physical fitness among soccer players [26,27,28], calf-muscle stretching can significantly benefit soccer performance [4]. The soleus muscle is one contributor to dorsiflexion ankle flexibility. It is a single-joint muscle located in the superficial posterior compartment of the leg that performs only plantar flexion action at the ankle joint. Additionally, along with the gastrocnemii muscles, the soleus muscle forms a muscle group (i.e., triceps surae) that is powerful in performing weight-bearing activities such as speed tasks [29] and jumping [30]. What is more, the soleus muscle is a major contributor to the stretch-shortening cycle of running [31], which is crucial for soccer athletes.

Previous studies on training have mainly focused on the effects of stretching on lower limb muscles, without considering the soleus muscle. For example, Akbulut et al. [21] studied the impact of an eight-week proprioceptive neuromuscular facilitation (PNF) stretching program on the kicking speed and range of motion in young male soccer players. They found that PNF stretching on the quadriceps, hamstrings, gluteus maximus, and calf muscles after a warm-up protocol increased kicking speed as well as hip and ankle range of motion. In addition, Huang et al. demonstrated that dynamic stretching exercises with a focus on the soleus muscle can increase soccer players’ ankle range of motion, dynamic balance, and performance speed [4]. However, Hernandez-Martinez et al. demonstrated that a general warm-up protocol and other stretching warm-up protocols focusing on quadriceps, hamstrings, glutes, and triceps had no significant impact on high jump, sprinting, or hitting the ball in soccer players. Here, the discrepancy in findings is obvious [32]. Moreover, Alipasali et al. reported that using a multiple-muscle-stretching approach with static and dynamic stretching techniques had a positive effect on sprinting performance in recreational male volleyball players [33]. However, there has been little research conducted on stretching the soleus muscle. Rezazadeh et al. showed that an eight-week functional stretching program for the gastrocnemius and soleus muscles reduced knee loading in the frontal and horizontal planes, indicating the effectiveness of functional stretching in enhancing knee joint biomechanics during walking [34]. 

Although the soleus muscle plays a crucial role in tasks such as running [31], most studies have focused on other lower leg muscles. While it is well-known that dynamic stretching can have positive effects when performed as a warm-up [11], there is limited knowledge about the long-term effects of this type of stretching. Therefore, our study aimed to investigate the long-term effects of adding dynamic soleus stretching to regular stretching exercises (3 × 30 s per muscle group for quadriceps, hamstring, and gastrocnemius) over a four-week period. We assessed ankle range of motion, soccer performance, and muscle thickness before and after the training period. Our hypothesis was that both the regular dynamic stretching protocol and the additional soleus stretching would result in improvements in the various tests. However, we further hypothesized that the added soleus stretching would lead to greater improvements compared to the regular dynamic stretching protocol.

## 2. Materials and Methods

### 2.1. Participants

The present study was a quasi-experimental research that focused on youth athletes in Iran’s Kerman province. The population consisted of 45 soccer players, and the sample size was determined based on previous studies by Barbosa et al. [16] and La Greca et al. [35] using G Power software (version 3.1.9.4; University of Kiel, Kiel, Germany). An α value of 0.05, power (1 − β) of 0.9, correlation coefficient of 0.5, and effect size of 0.28 were adopted. Forty-five healthy female youth athletes from provincial soccer teams volunteered for the study. The mean age of the participants was 22.98 ± 1.45 years, with a mass of 53.62 ± 2.69 kg and height of 169.60 ± 5.30 cm. The participants were randomly divided into three groups: regular dynamic stretch training (regular; n = 15), regular + soleus dynamic stretch training (regular + soleus; n = 15), and control group (controls; n = 15). All participants had at least three years of experience with soccer training (three sessions per week), and they were all right-leg dominant and had not sustained any lower limb injuries over the past six months [4]. Prior to data collection, all subjects provided written informed consent. Additionally, the participants completed an athlete injury questionnaire. The ethical approval for this study was provided by Shahid Bahonar University Ethics Committee (IR.UK.REC.1401.027) on 27 April 2022.

### 2.2. Procedure

After completing the consent form and demographic information, participants were randomly divided into three groups: regular, regular + soleus, and control. The study was conducted in a way that kept the purpose and the different types of stretching exercises blinded to the subjects. Participants performed stretching exercises for 4 weeks, with three sessions per week as part of their soccer exercises. The coach and one of the researchers (E.H.) monitored the participants’ stretching exercises during this time. Additionally, participants were asked to perform the ankle range of motion (ROM) test, drop jump test, and functional tests in random order before and after the 4-week stretching exercises. Notably, before stretching exercises and test instructions, video demonstrations were provided to the participant groups to familiarize them with the exercises. The regular stretching group participants were instructed to perform bilateral full-range dynamic stretches on the quadriceps, hamstrings, and gastrocnemius for three sets each, with a duration of 30 s, while participants in the regular + soleus group performed an additional soleus stretch for the same duration. The control group was asked not to stretch throughout the intervention period. All measurements from the subjects’ dominant leg were used in the data analysis [4].

### 2.3. Stretching Conditions

In this study, there were three conditions: regular stretching alone (regular group), regular stretching with additional soleus stretching (regular + soleus group), and no stretching (control group). In the regular group, the hamstring, quadriceps, and gastrocnemius muscles were bilaterally stretched three times each for 30 s using a dynamic stretching technique. For the regular + soleus group, participants were instructed to additionally stretch the soleus for 3 sets × 30 s. According to the literature review, there was a one-minute break after stretching all muscle groups. Participants in the control group did not stretch and were asked to sit down for 8 min instead. The hamstrings stretch was performed by standing on the ground with feet shoulder-width apart and bending the trunk forward with the goal of contacting the right foot with the left hand and vice versa with a dynamic movement. Additionally, the subject stretched their quadriceps while standing on one leg. After that, one hand was used to grab the foot that was lifted (stretching leg), and the heel was pushed towards the buttocks by bending the knee. In order to stretch the gastrocnemius, the individual took one step forward while standing, while the knee of the leg in the back was fully extended. The dynamic stretch was induced by pushing the front knee forward [4]. Lastly, the soleus muscle was stretched similarly to the gastrocnemius stretch except for using a wedge as well as by bending the knee. All participants were asked to perform the stretch until the point of discomfort, and in this protocol, each muscle group was stretched in 3 sets × 30 s at a velocity of 100 bits/minute (as fast DS) [36].

### 2.4. Measurements

#### 2.4.1. Ankle Range of Motion (ROM)

**Figure A1:**
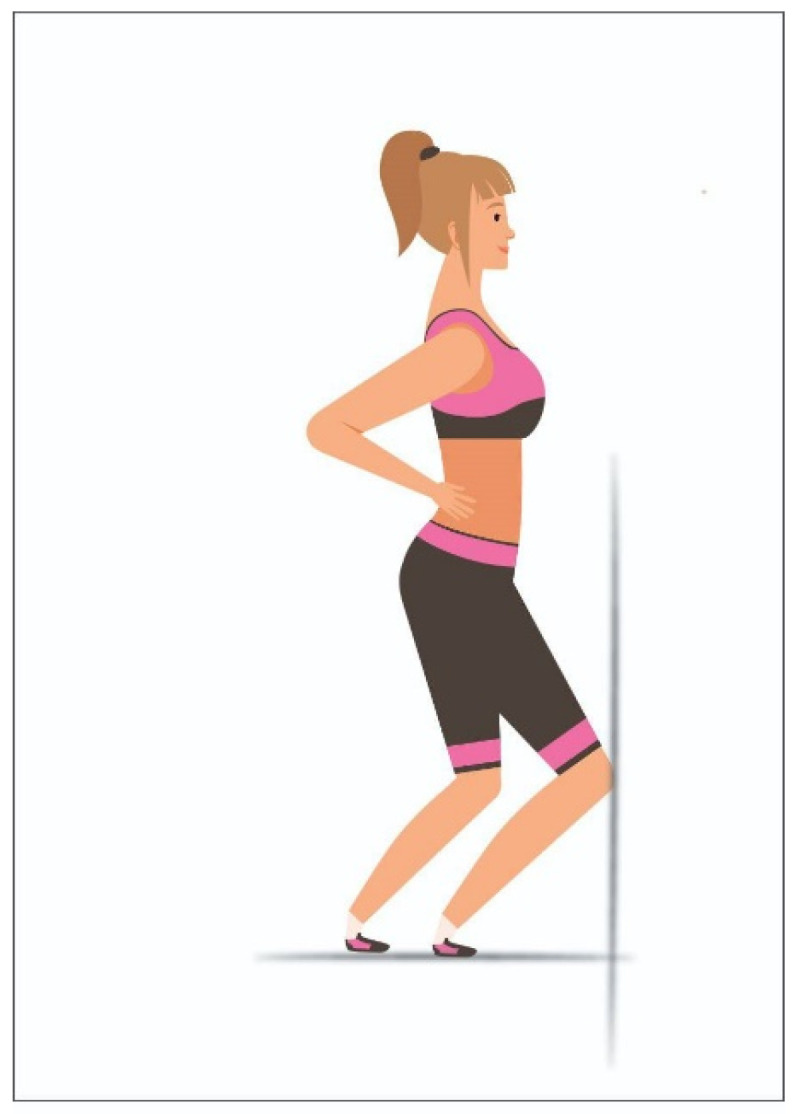
Ankle ROM test.

A reliable and consistent method for evaluating ankle range of motion (ROM) in a closed kinetic chain is the weight-bearing lunge test (Figure A1), as recommended previously [37]. To perform the test, participants were instructed to place their dominant foot on a measuring tape placed on the ground, positioning it 10 cm from the wall with the center of the middle toe and heel aligned with the tape. From this position, they were asked to lunge forward with their dominant limb, ensuring that their heel remained in contact with the ground while moving their knee forward until it reached the wall. This process was repeated until the greatest distance between their foot and the wall could be achieved while still touching the wall with their knee without lifting their heel off the ground. If this could not be accomplished, the foot was moved forward by 1 cm, and the process was repeated until the maximum distance between the foot and the wall could be attained while maintaining contact with the wall using the knee and keeping the heel on the ground. The interclass correlation coefficients (ICCs) for dorsiflexion (DF) ROM were 0.98 [37].

#### 2.4.2. Y-Balance Test

**Figure A2:**
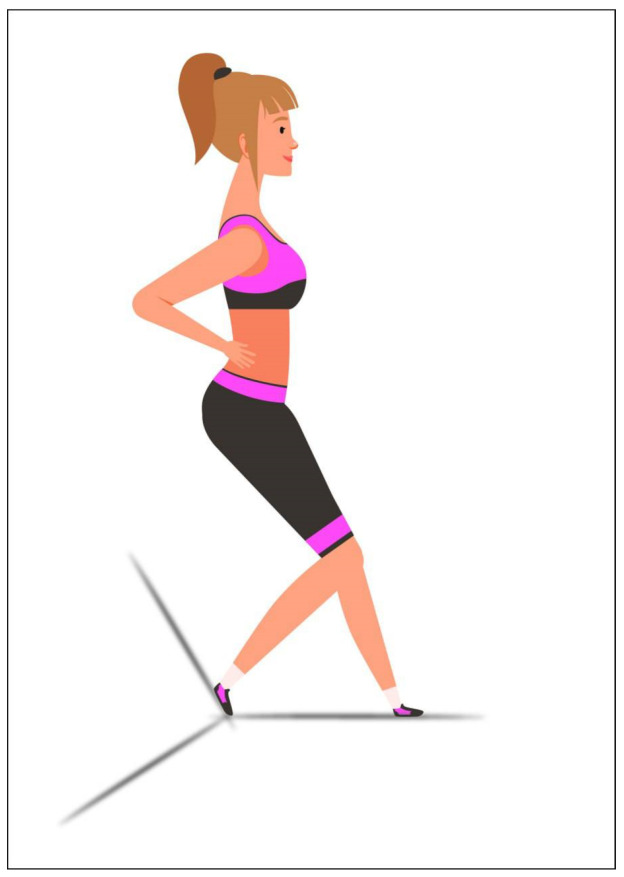
Y balance test.

The OctoBalance device (Check your Motion, Albacete, Spain) was utilized to assess dynamic balance by measuring three lower limb excursion directions: anterior (YBT-A), posteromedial (YBT-PM), and posterolateral (YBT-PL) (Figure A2). During the test, participants were instructed to stand on one leg in the center of the Y and extend their other leg as far as possible while maintaining balance and placing their hands on their hips. Participants were considered to have failed a trial if they touched the ground while reaching, lost balance during the movement, raised both arms to stabilize themselves, or lifted the heel of their standing leg during the execution [38]. Three trials were performed on each leg, with a passive recovery period of 10 s between them. The average outcome of the three trials for each leg was calculated for further analysis [39]. The mean and standard deviation for each direction were determined by using the range of values obtained from the test along with the actual length of the limbs measured for value normalization. To facilitate comparison between athletes, the range measurement was adjusted for limb length. The normalized value was determined by dividing the total of the three range values by three times the length of the limb and then multiplying it by 100 [40].

#### 2.4.3. Monitoring Tests (MTs)

##### Drop Jump (DJ)

**Figure A3:**
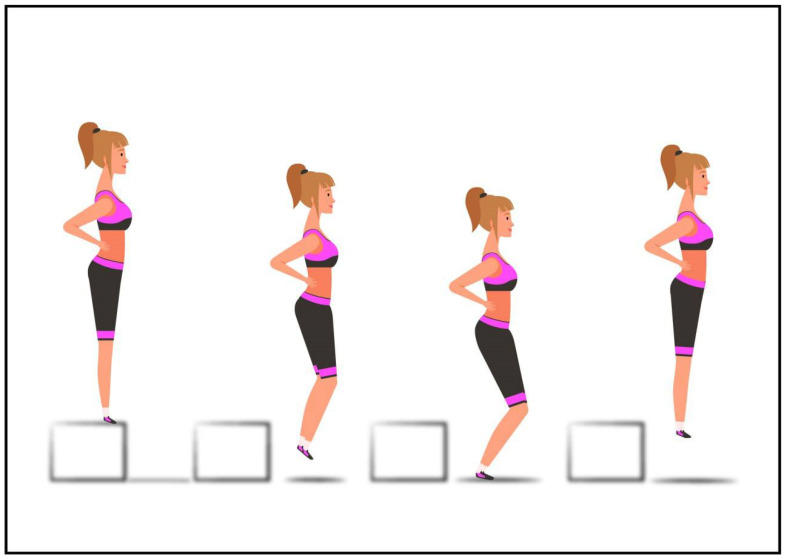
Drop jump test.

To begin the drop jump (DJ) test (Figure A3), participants stood on a 30 cm high box with their feet shoulder-width apart. The distance from the front of the box to a target line was determined by dividing each participant’s body height by two. Participants then jumped just beyond the target line, attempting to jump as high as possible after landing without any restrictions on arm motion [40]. Each participant performed the test three times, separated by a 45-s passive rest period, and the highest jump height was recorded for evaluation [39]. Three metrics were measured: jump height (determined by flight time), ground contact time (time spent on the ground between landing and takeoff), and RSI (flight time divided by ground contact time) [41]. The analysis of all recorded videos was conducted using the stable version of Kinovea (v. 0.8.15, Kinovea, Bordeaux, France) [42].

##### Dynamic Knee Valgus (DKV)

**Figure A4:**
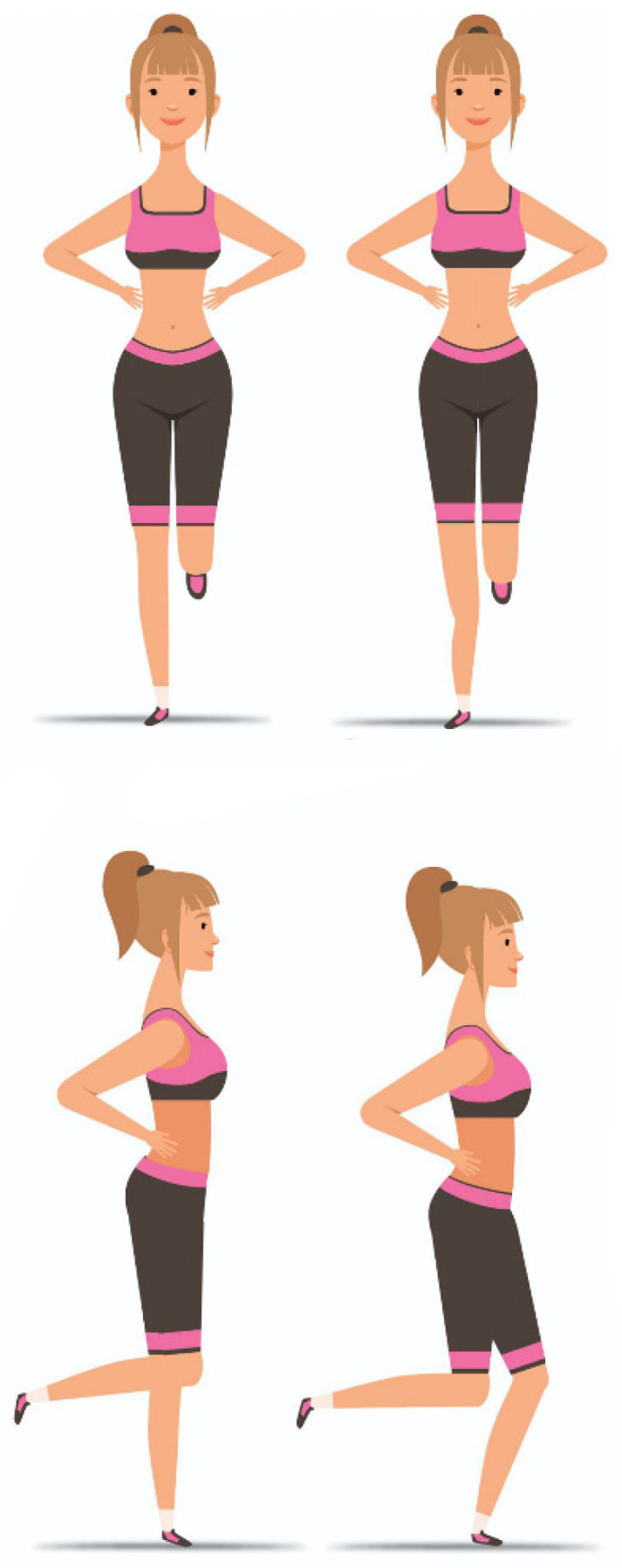
Dynamic knee valgus test.

To measure dynamic knee valgus (DKV) angles (Figure A4), the frontal plane projection angle (FPPA) was used. Two digital video cameras were employed—one positioned 2 m laterally and another 1 m in front of the subject, at knee level. The cameras included an EOS 5D Mark IV with Canon Log from the USA. Kinovea software (betaversion 0.9.5, Bordeaux, France) was used, along with free traffic monitoring software, which had been previously employed in investigations. Participants were instructed to stand on a step with the involved limb while maintaining a straight spine and placing their hands on their waist. They were then told to bend the knee of the involved limb until the heel of the uninvolved limb touched the ground, and immediately extend the knee of the involved limb to return to the starting position [37]. Anatomical markers were placed on the participants’ bodies to indicate reference points for lower limb alignment. The researcher determined FPPA during video analysis in the frontal plane projection by measuring the angle marked by the lines of the patella’s center, the upper anterior iliac spine, and a midline between the lateral and medial ankle malleolus. Anatomical landmarks, such as the greater trochanter, lateral condyle of the tibia, and lateral malleolus, were included in the sagittal projection analysis to determine the minimum angle of knee flexion (which should be at least 60° for a successful sample). The maximum angle of knee valgus was identified during the squat at the largest displacement from the initial position, and the starting angle of the test limb was calculated at the time of the single leg stance. If the researcher noticed any anomalies during the video analysis, that attempt was labeled a failure with no chance for repeat. The analysis was conducted using the average of three FPPA value attempts. A significant correlation between 2D and 3D analyses of lower limb alignment in the frontal plane projection angle was shown [37,43].

##### Illinois Agility Running Test

**Figure A5:**
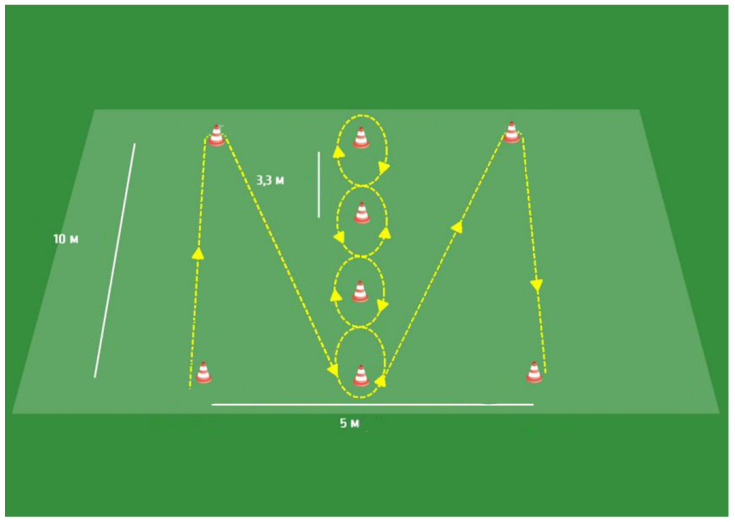
Illinois test.

The functional performance in this investigation was evaluated using the Illinois Agility Running Test (Figure A5), as described by Huang et al. [4]. and Michailidis et al. [44]. The test required a field with a width of 5 m and a length of 10 m, with four cones positioned 3.3 m apart in the middle of the area, marking the start, end, and two turning points. Participants were instructed to sprint as quickly as possible between the cones after the command “Go” was given. A successful trial was recorded if the participant completed the test without bumping into any cones and passed the finish line. The task was conducted on a soccer field, and two individuals were present at the start and finish lines to accurately record the starting and finishing times with the Q&Q stopwatch. Participants wore identical soccer equipment, including shoes and shirts, throughout the functional assessment. Three successful trials were completed with a 2-min rest period between each trial, and the best trial was selected for further analysis [4,45].

### 2.5. Data Analysis

The statistical analyses were performed using SPSS 26.0 software (IBM SPSS, Armonak, NY, USA). The normal distribution was evaluated using the Shapiro–Wilk test. A mixed-design analysis of variance (ANOVA) was applied to all variables, with a within-subject factor of time effect (pre-test and post-test), and a between-subject factor of group effect (regular, regular + soleus, and control) along with group–time interaction. If the group effect or interaction effect was found to be significant, a Tukey’s post hoc test was employed to detect potential differences. Effect size (ES, partial η^2^) was determined for all parameters, where a partial η^2^ of 0.02, 0.13, and 0.26 were defined as small, medium, and large, respectively. The significance level was set at *p* < 0.05, and the confidence interval was 95%.

## 3. Results

All variables displayed a normal distribution (*p* > 0.05). Mean and standard deviation of dependent variables before and after interventions are presented in Table 1. The results of mixed ANOVA, as shown in Table 1, indicate a significant difference in time effect (*p* < 0.05), group effect (*p* < 0.05), and group–time interaction (*p* < 0.05) for variables such as ankle-ROM, YBT, and DJ, and in the Illinois Agility Running Test. Although the time effect and group–time interaction for DKV were significant (*p* < 0.05), there was no significant difference in group effect (*p* > 0.05).

According to the results of the Tukey test (Table 2), there were significant differences (*p* < 0.05) observed between the regular group and the control group, the regular + soleus group, and the control group, as well as the regular group and the regular + soleus group in ankle range of motion (ROM) and drop jump (DJ) tests. Furthermore, in the Y-balance test (YBT), significant differences were noted between the regular group and the control group, as well as the regular + soleus group and the control group. In the Illinois Agility Running Test, a significant difference was observed between the regular + soleus group and the control group (*p* < 0.05).

## 4. Discussion

The aim of this study was to investigate the long-term effects on ankle joint range of motion (ROM), Y-balance, drop jump (DJ), and DKV, and Illinois agility running. Our results demonstrated that both intervention groups (regular and regular + soleus) showed significant improvements in ankle ROM, Y-balance, and DJ compared to the control group. However, only the regular + soleus group showed an improvement in the Illinois Agility Running Test. Athletes who performed additional soleus stretching had greater improvements in ankle ROM and DJ but not in DKV or Y-balance. Improved ankle ROM in the two stretching protocols may be related to either changes in muscle structure by decreasing muscle stiffness [46] or changes in stretch perception [47]. Huang et al. [4]. investigated the acute effect of added dynamic soleus stretching in a regular warm-up protocol and observed enhancement of ankle flexibility in the post-test. They suggested that adding dynamic soleus stretching into dynamic routine stretching was more effective in improving ankle ROM than DS routine. A similar superior effect with additional soleus stretching was seen after four weeks of stretch training in our current study. Conversely, some investigations have observed no changes in lower-extremity ROM as a result of an acute DS bout or even 12 weeks of DS training [17]. However, a previous meta-analysis reported that frequent DS can increase ROM in the long-term, although the effect size in static stretching and PNF stretching for an increase in ROM in the long-term was twofold higher [48]. Generally, the effects of long-term (chronic) DS, particularly the addition of soleus-muscle stretching to routine stretching protocols, on ankle ROM have rarely been studied. This demonstrates the significance of long-term research in this field.

The Y-balance test showed similar improvement in both intervention groups. Unilateral dynamic balance is especially critical for soccer players since most of their repetitive and explosive movements involve unilateral actions such as acceleration and deceleration tasks, rapid cutting maneuvers, kicking, jumping, and landing [49,50]. Therefore, poor unilateral dynamic balance can create unsafe situations for athletes and negatively impact their performance accuracy [50,51]. Studies that have utilized deep stimulation (DS) on the soleus muscle as a warm-up have revealed significant improvements in dynamic balance [4,52]. Kawaishi and Domen [53] found a direct relationship between the soleus H-reflex and increased postural difficulty, which may explain the improvements observed in the Y-balance test, at least in the regular + soleus group of the current study. In relation to the neural mechanisms that underlie this modulation, it has been proposed that a significant role is played by the attenuation of Ia afferents through presynaptic inhibition, which is regulated by descending commands. One potential benefit of this modulation of reflexes related to posture is the potential to mitigate joint oscillations that are mediated by reflexes, thereby shifting the control of movement towards higher centers responsible for movement coordination. The findings of this study indicate that individuals who were able to modulate the soleus H-reflex while performing various postural tasks exhibited a decrease in stretch reflex excitability. Moreover, these individuals demonstrated enhanced control over their posture during a dynamic balancing task. 

Female soccer athletes perform a variety of movement patterns during matches that put cumulative loads on their lower extremity joints and musculature, making it important to identify individuals with poor movement patterns in order to develop injury-prevention strategies [54,55,56,57]. Several interventions have been introduced to improve movement patterns in soccer players [1,54,58]. Previous studies have shown that female soccer players tend to have poorer landing and change-of-direction patterns compared to male players. For this reason, women soccer players incur anterior cruciate ligament (ACL) injuries at a rate significantly higher than that of their male peers. A significant proportion of anterior cruciate ligament (ACL) injuries in female athletes transpire in the absence of direct physical contact with another player. Non-contact injuries can occur due to inadequate biomechanics during physical activity and a deficiency in neuromuscular control [59,60]. In the current study, both stretching conditions resulted in improved drop jump (DJ), with the regular + soleus group showing greater improvement compared to the regular group. However, there was no significant difference between the groups in the dynamic knee valgus (DKV) test, which was not consistent with previous warm-up stretching studies. For example, Avedesian et al. [6]. reported an improved landing strategy and increased hip adduction angle after combined static and dynamic warm-ups, while a literature review by Lima et al. [61]. suggested that reduced dorsiflexion range of motion is associated with increased dynamic knee valgus. Therefore, interventions such as flexibility programs to improve dorsiflexion range of motion can be useful in enhancing movement quality. In the present study, both dynamic protocols had positive effects on DJ but not on DKV. Therefore, although performance can be improved to a greater extent with additional soleus stretching, it may not decrease DKV.

Considering the Illinois Agility Running Test, our results showed a positive progress in regular + soleus stretching protocols when compared to the controls. These results are in line with studies of dynamic stretching warm-up protocols [62,63]. Ishak et al. [62] indicated a dynamic warm-up protocol enhanced the repeated sprint test (six sets × 30 m-sprint) in male university team-sport athletes. The observed results of Dallas et al. [63] also revealed that DS provide beneficial effects for improvement of performance tests such as sprint tests and the *t*-test. Additionally, considering the soleus muscle, Huang et al. [4] reported an improved sprint performance, however, they found no difference between the regular and soleus stretching group. According to this evidence additional soleus stretching might be beneficial in the long-term but is likely not relevant when considered as a warm-up.

This study had some limitations that should be considered. Firstly, our sample size was small and to generalize these results to female soccer players it is recommended to conduct similar research with a larger sample size. Secondly, the duration of the study (i.e., 4 weeks) was quite short. Therefore, it is suggested that the effects of soleus stretching should be investigated over a longer period. Additionally, follow-up tests over a prolonged duration might be considered in future studies. Thirdly, the physiological effects of stretching were not been investigated in this research. It is suggested to investigate these limitations in future research.

## 5. Conclusions

The results of the study confirm that long-term (4 weeks) soleus stretching leads to significant improvements in ankle range of motion, Y-balance, DJ, and the Illinois Agility Running Test. Therefore, if soleus stretching is added to lower limb dynamic stretching in the long term, it could have a positive influence on the skills and performance of female players. Therefore, trainers are recommended to incorporate dynamic stretching into their training sessions and warm-ups to improve player abilities.

## Figures and Tables

**Table 1:** The records of studied groups in pre-test and post-test.

	Groups	Regular Mean (SD)		Regular + Soleus Mean (SD)		Controls Mean (SD)		Time Effect	Group Effect	Group × Time Interaction
Variables		Pre-Test	Post-Test	Pre-Test	Post-Test	Pre-Test	Post-Test			
Ankle-ROM (cm)		9.36 (0.22)	9.79 (0.18)	9.43 (0.19)	10.29 (0.62)	9.33 (0.27)	9.13 (0.29)	F = 33.99 *p* = 0.001 * ES = 0.44	F = 21.67 *p* = 0.001 * ES = 0.50	F = 24.19 *p* = 0.001 * ES = 0.53
YBT (cm)		78.60 (3.33)	84.38 (2.94)	78.65 (2.54)	86.35 (2.20)	77.83 (2.33)	78.20 (2.09)	F = 114.15 *p* = 0.001 * ES = 0.73	F = 17.50 *p* = 0.001 * ES = 0.45	F = 25.79 *p* = 0.001 * ES = 0.55
DJ (RSI)		0.86 (0.04)	0.92 (0.03)	0.83 (0.06)	0.90 (0.02)	0.91 (0.02)	0.92 (0.02)	F = 30.89 *p* = 0.001 * ES = 0.42	F = 8.11 *p* = 0.001 * ES = 0.27	F = 12.69 *p* = 0.001 * ES = 0.37
DKV (°)		18.39 (4.26)	16.31 (3.65)	19.07 (2.67)	16.05 (2.57)	19.17 (5.86)	19.70 (5.81)	F = 58.50 *p* = 0.001 * ES = 0.58	F = 1.06 *p* = 0.35 ES = 0.04	F = 28.50 *p* = 0.001 * ES = 0.57
Illinois (s)		18.51 (4.73)	14.96 (3.86)	16.28 (3.54)	13.79 (2.90)	17.79 (4.04)	18.96 (2.51)	F = 23.11 *p* = 0.001 * ES = 0.35	F = 3.41 *p* = 0.042 ES = 0.14	F = 17.86 *p* = 0.001 * ES = 0.46

**Note:** ROM = range of motion; YBT = Y-balance test; DJ = drop jump; DKV = dynamic knee valgus; ES = effect size; * = *p* < 0.05.

**Table 2:** Comparison of studied variable within the three groups.

Variables	Groups	*p*	CI
Ankle-ROM (cm)	Regular − control	0.002 *	(0.11)–(0.58)
	Regular − regular + soleus	0.014 *	(−0.51)–(−0.049)
	egular + soleus − control	0.001 *	(0.39)–(0.86)
YBT (cm)	Regular − control	0.001 *	(1.54)–(5.40)
	Regular − regular + soleus	0.42	(−2.93)–(0.92)
	Regular + soleus − control	0.001 *	(2.55)–(6.41)
DJ (RSI)	Regular − control	0.036 *	(−0.050)–(−0.001)
	Regular − regular + soleus	0.046 *	(0.001)–(0.049)
	Regular + soleus − control	0.001 *	(−0.075)–(−0.026)
DKV (°)	Regular − control	0.38	(−5.89)–(1.73)
	Regular − regular + soleus	0.99	(−4.02)–(3.60)
	Regular + soleus − control	0.46	(−5.68)–(1.94)
Illinois (s)	Regular − control	0.41	(−4.74)–(1.45)
	Regular − regular + soleus	0.38	(−1.40)–(4.79)
	Regular + soleus − control	0.032 *	(−6.44)–(−0.23)

**Note:** * = *p* < 0.05; CI = confidence interval.

## Data Availability

The data that support the findings of this study are available from the corresponding author, upon reasonable request. The data are not publicly available due to containing information that could compromise the privacy of research participants.
